# Orchestrating explainable artificial intelligence for multimodal and longitudinal data in medical imaging

**DOI:** 10.1038/s41746-024-01190-w

**Published:** 2024-07-22

**Authors:** Aurélie Pahud de Mortanges, Haozhe Luo, Shelley Zixin Shu, Amith Kamath, Yannick Suter, Mohamed Shelan, Alexander Pöllinger, Mauricio Reyes

**Affiliations:** 1 ARTORG Center for Biomedical Engineering Research, University of Bern, Bern, Switzerland; 2 Department of Radiation Oncology, Inselspital, Bern University Hospital, University of Bern, Bern, Switzerland; 3 Department of Diagnostic, Interventional and Pediatric Radiology, Inselspital, Bern University Hospital, Bern, Switzerland

**Keywords:** Translational research, Data integration, Machine learning, Image processing

## Abstract

Explainable artificial intelligence (XAI) has experienced a vast increase in recognition over the last few years. While the technical developments are manifold, less focus has been placed on the clinical applicability and usability of systems. Moreover, not much attention has been given to XAI systems that can handle multimodal and longitudinal data, which we postulate are important features in many clinical workflows. In this study, we review, from a clinical perspective, the current state of XAI for multimodal and longitudinal datasets and highlight the challenges thereof. Additionally, we propose the XAI orchestrator, an instance that aims to help clinicians with the synopsis of multimodal and longitudinal data, the resulting AI predictions, and the corresponding explainability output. We propose several desirable properties of the XAI orchestrator, such as being adaptive, hierarchical, interactive, and uncertainty-aware.

## Introduction

As artificial intelligence (AI)-based support systems for radiology become more widely available in clinical practice, limitations arising from their “black box” nature lead to increased enunciation of the need for explainable AI (XAI)^1,2^. Interpretable or explainable machine learning and AI algorithms are systems where a human user can understand how the prediction (output) is reached based on the input^3^. The terms “interpretable” and “explainable” are often used interchangeably, but some authors emphasize the distinction between the terms^4^. In this narrative review, we will use the term “explainable” as proposed by Graziani et al. They defined “explainable” as “[…] to *illustrate* what features or high-level concepts were used by ML [machine learning] system to generate predictions for one or multiple inputs.”^4^ Ultimately, in clinical practice, XAI is meant to serve a common purpose – providing insight into AI models to enhance physician’s efficacy and patients’ safety. Explainability can be achieved through a variety of different XAI methods; for example, in medical image analysis, XAI is most commonly based on visual explanations, so-called “saliency maps”.

XAI systems offer a variety of advantages over “black box” models by exhibiting better quality assurance and auditability, as well as increased user trust in the system^5^. Yet some challenges are so far unmet and impede the tapping of XAIs’ full potential. These include the lack of studies that enrich radiological XAI systems with other types of clinical data (multimodal XAI) or use longitudinal data sets. Merging these data types and deriving a meaningful overall explanation is challenging and has received little attention. We postulate that further developments of multimodal and longitudinal XAI are essential and vastly needed in many clinical workflows^6,7^.

In this narrative review, we aim to inform readership from biomedical engineering and informatics disciplines, medical doctors, and other healthcare professionals about multimodal data fusion and longitudinal data analysis for XAI. In addition, in light of the current developments of large language models, we propose the “XAI Orchestrator” as an instance, or virtual assistant to doctors, which is capable of coordinating, organizing, and verbalizing explanations of specific AI models and provide a user-centered mechanism for doctors to further enquire AI models operating on multimodal and longitudinal data.

## XAI for multimodal and longitudinal data

In healthcare, diagnoses and treatment decisions are rarely based on a single scan or blood draw - they are made in the synopsis of all relevant information available^8^. A majority of radiologists (87%) stated in a survey that clinical information impacts image interpretation significantly^9,10^. This clinical information can include text-based data such as a transcript of patient-reported disease history, findings from physical exams, vitals, laboratory measurements, and, less frequently, complex -omics data such as genomics. Combining these different data types, hereafter referred to as multimodal data, for deep learning tasks is a promising and increasingly popular approach^11–13^. AI systems can profit significantly from assimilating multimodal data into prediction and classification models to imitate integrative human clinical decision-making. This can boost their robustness and accuracy, enable the discovery of new biomarkers and therapeutic targets^6,14^, as well as improve model performance^15–17^.

Similarly, knowledge about the temporal evolution of biological processes plays a crucial role in health care. For example, in oncology, longitudinal information is important to assess slowly progressive forms of cancer or cancers with yet unclear dignity^18^ (benign vs. malignant), as well as in the evaluation of treatment response. Just as for multimodal data, introducing explainability methods for the analysis of longitudinal data may contribute to the systems’ stability, robustness, and confidence^19^.

## Discussion of previous work on XAI for multimodal data

Multimodal fusion has various benefits over the use of a single modality. Multiple modalities can enable the visualization of complementary information, enhance prediction robustness, and allow a system to make predictions even when one modality is missing^20^. Radiological data has been combined with other data types for predictive AI systems in various clinical disciplines like oncology^21–24^ or neurology^25,26^. For a systematic review of studies on the fusion of medical imaging and electronic health record (EHR) data using deep learning, we refer the reader to Huang et al.^10^. Yet oftentimes, different research groups investigate similar questions with variable approaches and differing results. For example, the prediction of Mild Cognitive Impairment or Alzheimer’s disease based on the ADNI dataset is frequently investigated^27–32^. But their predictive accuracy varies, and many studies do not discuss which input modalities or features contributed most to the prediction. This makes comparisons among the studies difficult and limits the transferability of results. Beyond model comparison at the level of performance, XAI techniques could enhance comparison regarding pathophysiological plausibility by providing influential features, for example, the volume of the hippocampus and amygdala as biomarkers of cognitive impairment^33^.

Currently, only a few of these studies on multimodal AI have made an effort to make their systems explainable, even though the importance of multimodal XAI systems has been highlighted^6^. Currently, one of the most comprehensive studies on multimodal XAI is by Soenksen et al., who developed the “Holistic AI in Medicine (HAIM)” framework, for combining imaging, tabular, text, and time series data^16^. The authors propose modality-specific embeddings, which are combined and fed into an eXtreme Gradient Boosting (XGBoost) classifier to perform a variety of prediction tasks. When the authors tested their framework in over 14’000 different prediction models, they found that predictions based on multimodal data outperform unimodal comparators by 6-30%. For interpretability, Shapley values were calculated for all input data^16^. This study laid a great foundation; for further improvement, development and testing of (X)AI systems also need to be performed on datasets featuring levels of data quality as found in daily clinical routines, additionally to using well-curated research datasets. Also, the data acquired since admission may not be sufficient to acknowledge all relevant information, especially in chronic diseases. Systems should be aimed at incorporating data from earlier hospital stays and outpatient consultations. Furthermore, modeling outcomes in the form of binary classification tasks does not fully capture clinical practice. For XAI, the complexity of multi-class or multi-label problems is also increased with respect to binary classification problems. Finally, the evaluation of a multitude of different models composed of permuted combinations of input features is suitable for the initial validation of a proposed framework. Afterwards, it is important to test with a small, carefully selected number of models that address clinically relevant questions.

Another recent example for the successful combination of imaging with other data types for XAI is a study by Taleb et al.^15^. They introduce a self-supervised learning approach where retinal fundus images were combined and aligned in the feature space with different types of genetic data using a contrastive loss. In this study, the authors adapted gradient-based explainability algorithms to understand cross-modal associations. The authors showed that image model performance was improved considerably by including genetic information. Yet genetic analyses are often costly and time-intensive to obtain. Prior to resorting to high-effort data modalities, it would be desirable to predominantly incorporate readily available clinical data, such as patient demographics, medical history, vitals, and routine laboratory values. Additionally, clinical applicability needs to be always kept in mind during development. While a prediction of cardiovascular risk factors such as age, sex, smoking status, blood pressure, and BMI from retinal fundus images is a technically interesting task, this information could also be obtained with a brief patient visit.

Finally, Cao et al. predicted colorectal cancer microsatellite instability (MSI) from histopathological whole slide images (WSIs)^34^. The prediction was based only on a single type of data, the WSIs, but other data types were used to enable interpretability of the model. The authors extracted the pathological signatures that contributed most to the prediction of MSI and explored their correlation to genetic and transcriptomic patterns, such as patterns relating to deficient deoxyribonucleic acid (DNA) repair and immune activation.

Other studies exist that have combined multimodal data for XAI systems but did not involve medical images. For example, Jurenaite et al. used non-fixed sets of mutated genome sequences (mutomes) and transcriptomes in a transformer-based deep neural network, aiming to predict seven common tumor types^35^. For explainability, primary attribution methods were applied to obtain omic-specific attribution scores per patient and feature type. For the genetic data, the authors reported that the genes with the highest attribution scores all carried known biological significance in cancer occurrence, which provides valuable confirmatory evidence on the reliability of the AI system. In Prelaj et al., the efficacy of immunotherapy in non-small cell lung cancer was predicted based on demographics, laboratory measurements, tumor characteristics and staging, treatment information, and radiological information^36^. The radiological features consisted of information on whether certain types of metastases were present; no imaging data was fed directly into the model. For explainability, they used SHAP, which demonstrated that the most relevant features in their model are clinical biomarkers that have previously been shown to be important^36^.

There are multiple toolkits, such as AIX-360^37^, Alibi^38^, Captum^39^, EthicalML-XAI^40^, iNNvestigate^41^, Quantus^42^, among others, offering readily implemented XAI methods for a wide variety of tasks applicable to medical imaging (Table 1). While many of these libraries can process multiple input data types separately, only Captum explicitly offers multimodality for the joint processing of input features stemming from different data types. To facilitate quality control and comparability, some of the toolkits also offer XAI evaluations^37,39,42^.

**Table 1:** Overview of current XAI libraries and their supported input data types

Library	Images	Text	Tabular	Audio	Video	Longitudinal	Evaluations
AIX-360^37^	✓	✓	✓	✗	✗	✗	✓
Alibi explain^38^	✓	✓	✗	✗	✗	✗	✗
Captum^39^	✓	✓	✓	✓	✓	✗	✓
DALEX^92^	✗	✗	✓	✗	✗	✗	✗
EthicalML-XAI^40^	✗	✗	✓	✗	✗	✗	✗
H2O^93^	✗	✗	✓	✗	✗	✗	✗
iNNvestigate^41^	✓	✓	✓	✗	✗	✗	✗
InterpretDL^94^	✓	✓	✗	✗	✗	✗	✓
PAIR saliency^95^	✓	✗	✗	✗	✗	✗	✗
Quantus^42^	✓	✓	✓	✗	✗	✓	✓
Shapash^96^	✗	✗	✓	✗	✗	✗	✓
Tf-explain^97^	✓	✗	✗	✗	✗	✗	✗
Torch-cam^98^	✓	✗	✗	✗	✓	✗	✓
TorchRay^99^	✓	✗	✗	✗	✗	✗	✓
Zennit^100^	✓	✗	✗	✗	✗	✗	✗

The supported input data types were assessed by screening the library/package documentation and the provided examples. If the email address of the main developer of the library was available online, we also reached out to them to confirm the supported data types. If no answer was received, the assessment was based on the online documentation only. References cite the corresponding publication or the GitHub account in the absence of a publication. Versions as of end of 2023.

## Challenges of XAI for multimodal data

Some challenging aspects need to be considered when designing XAI that is supposed to handle multimodal data:

1. *Choice of XAI method*. Saliency maps suited for radiological data might not be applicable for other data types, such as tabular data^43^. Currently, many studies use early fusion techniques, where data from different modalities are prematurely combined or concatenated. This makes it challenging to understand to *what extent*, *where-in* and *how* each modality contributes to the system’s decision.
2. *Domain knowledge*. Some -omics data, like metabolomics, are intrinsically complex, and interpretation should be performed by a trained expert. Developers of XAI systems and users can only be experts in some domains of human medicine. As the amount and type of information per patient increase, multi-modality AI systems are expected to emerge, leading to an amplification of the black-box nature of AI systems.
3. *Curse of dimensionality*. With increasingly sophisticated -omics technologies, the dimensionality of data increases rapidly, thereby surpassing the number of cases, which remains similar over time. This phenomenon is described as the “curse of dimensionality”^44^. The high dimensionality of data that makes it attractive to research may, at the same time, be a rate-limiting factor in the development of algorithms capable of generalizing to real-world scenarios^45^. In this situation, XAI becomes crucial as interpretability methods can help to find and eliminate spurious correlations and shortcut learning^46–48^.
4. *Susceptibility to adversarial attacks*. The robustness of multimodal models is a topic of ongoing discussion because multimodal models may be equally or even more vulnerable to adversarial attacks than models using a single modality. This susceptibility to adversarial attacks results from the negative impact of increasing input dimensions on adversarial robustness^49–51^.

Additional organizational or technical challenges regarding multimodal machine learning and AI in healthcare have previously been pointed out^20,44,52^.

## Discussion of previous work on XAI for longitudinal data

Regarding the combination of longitudinal image data with other data types, Rahim et al. aimed to predict Alzheimer’s Disease from three-dimensional (3D) magnetic resonance imaging (MRI) data with three time points, in combination with non-imaging data^53^. They suggest using a 3D convolutional neural network to learn the deep spatial and inter-slice features from the MRI volumes for every time point and a bidirectional recurrent neural network to learn the inter-volume temporal features between time points. Additionally, they provide two types of visual explanations: activation maps of two-dimensional (2D) MRI slices from each time point and 3D brain surface rendering.

Besides the study by Rahim et al. not many are leveraging longitudinal radiological images for an XAI system. More progress has been made in other non-imaging fields. For example, longitudinal gene expression data from a dietary intervention study was used by Anguita-Ruiz et al. to analyze temporal gene-gene relationships^54^. With a sequential rule mining algorithm, they aimed to find biologically relevant patterns and present them in an easily understandable format. Shashikumar et al. used longitudinal data from EHRs for early sepsis prediction in intensive care patients^55^. Additionally to the prediction, the system also provides local interpretability by outputting the top factors contributing to the individual risk of sepsis for every patient at every time point. In Ibrahim et al., the authors evaluated a longitudinal dataset of electrocardiograms in combination with age and sex to predict acute myocardial infarction^56^. They devised three algorithms, of which an XGBoost model attained the best performance. Shapley values were calculated, and age, age-adjusted Charlson Comorbidity Index, and duration of the QRS complex were shown to contribute most to the prediction. For an overview of XAI methods that can be applied to time series data not specific to medical imaging, we refer the reader to Rojat et al.^19^.

As for multimodal XAI, studies involving radiological data are lacking. It has been suggested that research on XAI for longitudinal data is scarce because the input (single or collective time points) often lacks meaningful interpretation to humans^57^. In our opinion, this is not always true. In the medical field, certain input information becomes meaningful *only* in combination with preceding or subsequent data. For example, for the laboratory diagnosis of acute myocardial infarction (AMI), high-sensitivity cardiac troponin (hs-cTn) needs to be measured at least twice^58^. AMI is diagnosed if hs-cTn is elevated over the 99^th^ percentile of a healthy reference group in at least one measurement and an increase or decrease in hs-cTn is observed between measurements. This allows to distinguish AMI-related elevations from chronic conditions such as chronic kidney disease^58^.

## Challenges of XAI for longitudinal data

Just as for multimodal data, integrating time series of images into XAI models, potentially combined with other types of data, poses some challenges that need to be considered.

**Fig. 1:**
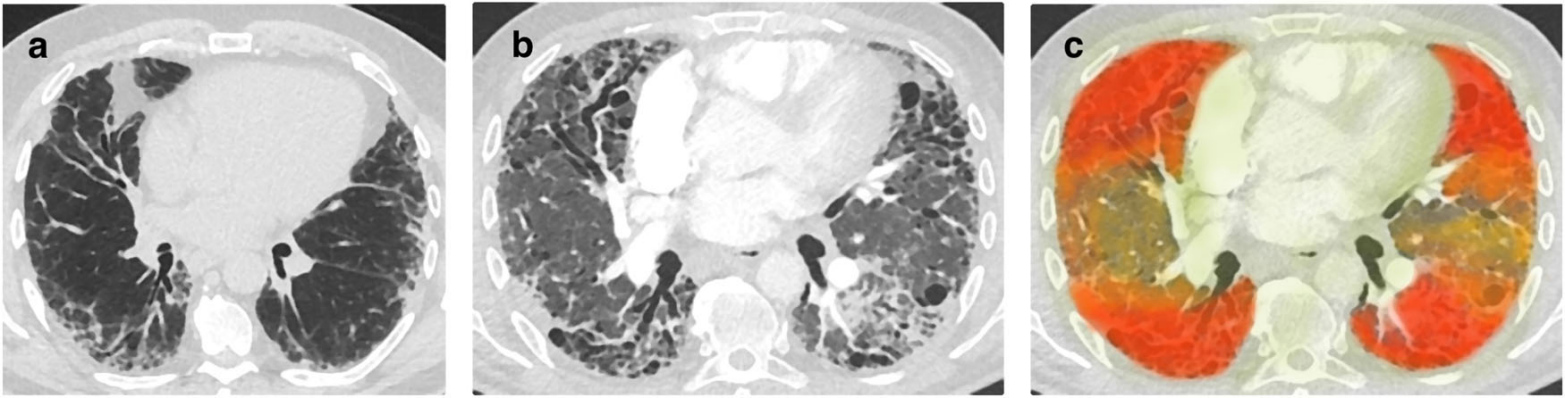
Improving over current saliency maps for longitudinal scenarios. The delta saliency map. In this example case of interstitial pulmonary fibrosis, the left image (**a**) was taken around two years prior to the middle image (**b**). During the two years, the disease progressed heavily. The delta saliency map (**c**) shows this disease progression through the yellow, orange, and red color overlays. The frontal and dorsal areas of the lungs, which are heavily affected, as well as the subpleural areas, are expected to contribute most to the classification and are therefore overlayed with the highest opacity of color, whereas the extrapulmonary areas are only lightly overlayed as they are expected to contribute only marginally.

1. *Continuous vs. intermittent recording of data*. Most radiological images are acquired intermittently. Ultrasound, on the other hand, allows recording images continuously over time, thereby capturing mechanistic information, such as heart chamber contractions and blood flow in echocardiography. For such continuous data, the development of XAI techniques that are also temporally-based, such as video sequences of color-coded saliency information, could lead to improved intelligibility of the underlying temporal information.
2. *Data sparsity and sampling intervals*. Although data imputation techniques aim at filling missing values with interpolations of adjacent measurements, such approaches are not always useful depending on the underlying physiology of the parameters. For example, prostate-specific antigen (PSA) evolves steadily over time, so if it is measured twice within several months, the actual values for the period most likely lie around these two measurements. Yet other parameters reflect acute fluctuations for which the sampling interval needs to be flexible. For example, two C-reactive protein (CRP) measurements, taken several months apart, may both show normal values of <3 mg/L, while the patient could have developed and recovered from severe pancreatitis, with CRP of say, 280 mg/L in between. With respect to multimodal data, the more data types are involved, the more difficult it is to define meaningful sampling intervals.
3. *Representation of spatio-temporal relationships*. In clinical workflows, the spatio-temporal relationships in imaging are important. However, current saliency maps show *where* an AI system focuses on and are limited to working with single time points. If a patient undergoes imaging multiple times for the same disease, it would be desirable for a saliency map to reflect the extent of the disease, implicitly characterizing disease information about the “*location*” and “*extent of progression*”. We therefore propose a “*delta saliency maps*”, which would color-code imaging patterns on disease evolution status (e.g, disease progression, response to therapy, stable disease, etc.), while the opacity of such a map would reflect how important (i.e., attribution level) that local area is to the final diagnosis of the explained AI system. (cf. Fig. 1).

## Proposing the XAI orchestrator

**Fig. 2:**
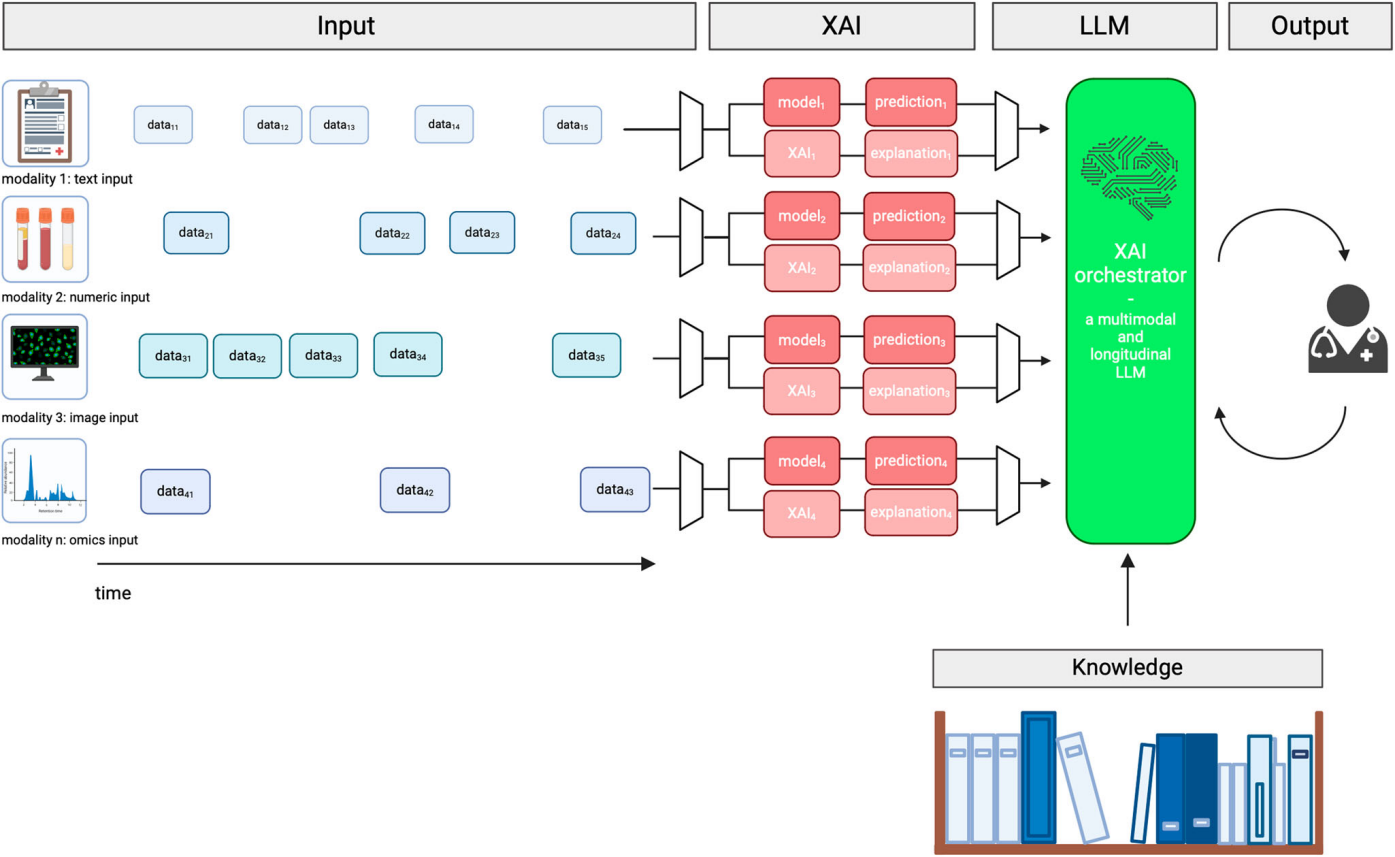
Conceptual description of the XAI orchestrator. Clinical guidelines and recent research, constitute the knowledge base of the XAI Orchestrator. Additionally, multimodal patient-specific data is collected. After outcome prediction, XAI methods are applied to generate modality-specific or time-specific explanations. The superordinate XAI orchestrator aggregates all information and generates a comprehensive overall explanation while enabling further inquiries by an expert. Figure created with BioRender.com.

Considering the increased complexity of multimodal and longitudinal XAI, as well as the need for the combination of both, we propose the XAI orchestrator. Its development is motivated by oncological tumor boards where specialists from different medical fields share their expertise, discuss test results, and combine their findings to select an optimal treatment strategy. We imagine a similar approach for an XAI system: Pretrained biomedical knowledge, as well as patient-specific multimodal and longitudinal data, are collected and used to predict an outcome. XAI systems interpret the results, providing modality-specific explanations. Subsequently, everything is assembled by a superordinate, Large Language Model (LLM)-based *XAI orchestrator*, which considers the input data, the prediction, and the explainability output (cf. Fig. 2). It produces a user-friendly overall explanation and answers follow-up questions. Here, we do not provide a full implementation and results of the XAI orchestrator but describe how it could arise from the current developments of LLMs as well as its desirable properties, functionalities, and metrics. In the supplementary materials (Supplementary Discussion A with Supplementary Fig. 1 and Supplementary Discussion B with Supplementary Fig. 2), we provide two clinical case examples of diagnostic processes where multimodal and longitudinal data are essential to illustrate situations in which the XAI orchestrator could be employed.

## The XAI orchestrator and LLMs

LLMs have many potentially beneficial applications in healthcare practice and research, including diagnostic (e.g., prediction of disease risk and outcomes) and procedural (e.g., streamlining of clinical workflows, documentation, cost-effectiveness) tasks^59^. Recently, multiple language models specific to the biomedical domain have been released, for example, models of the BERT family. BioBERT was pre-trained on PubMed abstracts and PubMed Central full-text articles and exceeded previous models in tasks like named entity recognition, relation extraction, and question answering^60^. Med-BERT was pretrained on structured EHR data from over 28 million patients and evaluated on the prediction of pancreatic cancer, and heart failure in patients with diabetes^61^.

Although the main strength of LLMs lies in the processing of and responding to text input and in logical reasoning, strategies to leverage LLM’s capabilities for image analysis are being investigated. For example, Wang et al. propose ChatCAD, a system that takes Chest X-rays as input, and passes them to different computer-aided diagnosis systems, which produce vectors of output^62^. These vectors are translated into text, concatenated, and passed to an LLM, which analyzes them jointly, incorporates pre-trained medical knowledge, and summarizes the results.

Currently, many research groups also work on LLMs that combine multiple medical data types. GLoRIA is an attention-based framework that learns global and local medical imaging representations from radiology reports by contrasting text parts with image sub-regions from their paired chest x-rays^63^. To address the scarcity of publicly available image-report pairs, compared e.g. to the number of accessible images of cats and dogs, MedCLIP uncouples images and texts for multimodal contrastive learning, thereby increasing the number of training data and mitigating the problem of false negative reports (i.e. many reports do not belong to the target patient’s images, yet may still correctly describe their findings)^64^. In MedKLIP, the authors developed a triplet extraction module that encodes medical entities extracted from radiology reports, their position, and presence or absence as a triplet. This triplet is then encoded with an entity translation that provides detailed descriptions of entities by querying a medical knowledge database.

Even the capabilities of non-medicine-specific models are tested: Although Open AI states that GPT-4V is not suitable for the interpretation of medical images^65^, its performance on multimodal medical images with or without other types of clinical data has been evaluated^66^. While it can distinguish between image modalities and recognize anatomical regions, its diagnostic capabilities are currently suboptimal for clinical use, illustrating the importance of dedicated training on medical data.

We believe that an LLM-based orchestrator could be beneficial in XAI for clinical settings as it could provide a verbalization of explanations adapted to the current user and situation. Moreover, LLM-based technologies could enable a bidirectional “dialogue” between users and (X)AI systems. In the more or less distant future, such systems may serve as a virtual assistant capable of working as a counselor in clinical scenarios.

## Desirable properties, functionalities, and metrics of the XAI orchestrator

### Properties

From a clinical point of view, we propose the following attributes for the XAI orchestrator to be helpful in daily practice (Table 2):

**Table 2:** Summary of the proposed properties, functionalities, and metrics of the XAI orchestrator

Properties	Functionalities	Metrics
Adaptive Hierarchical Uncertainty-aware Interactive Time effective Causality- and Codependency-aware Modular Privacy-preserving	Information fusion Task triaging Scenario simulation	Faithfulness Robustness Localization Complexity Randomization Axiomatic metrics

1. *Adaptive*. The XAI orchestrator must cope with a varying set of potentially sparse input data. If underlying data contains complementary rather than mutual information, explanations should improve^7^. To enable such adaptivity, the XAI orchestrator needs to be evaluated on representative real-world data.
2. *Hierarchical*. The XAI orchestrator should be able to provide explanations at various levels of detail, with further information being available on request.
3. *Uncertainty-aware*. The XAI orchestrater should also consider the quality of the underlying data, regarding completeness, recency, noise level, etc., and weigh their respective XAI outputs accordingly in the overall explanation.
4. *Interactive*. The XAI orchestrator should comprise a chat mode. Virtual reality equipment could facilitate immersive and flexible interaction tailored to the user’s preferences.
5. *Time effective*. The XAI orchestrator should be integrated with time-effectiveness in mind since it was found that clinicians sometimes prefer rapid, less detailed information^67^.
6. *Causality- and Co-dependency aware*. It would be desirable for the XAI orchestrator to be aware of co-dependencies and causalities in the data, regarding both causality of biological processes, as well as “meta-causality” relating to iterative ordering and evaluation of diagnostic testing. Explicit knowledge of causal relationships is mostly unleveraged, as contemporary (X)AI consists mostly of deep learning systems relying on correlations between input and outcome variables. Nevertheless, causality has recently enjoyed increasing attention again, with discussion about causality in deep learning^68^ and medical imaging^69,70^.
7. *Modular*. The different models and XAI methods that the orchestrator is composed of should allow for flexible, modular testing and validation. This would facilitate targeted updating and maintenance in the case of a data shift, i.e., the image processing unit can be revised after the introduction of a new scanner without the need for retraining of parts unaffected by the data shift.
8. *Privacy preserving*. The XAI orchestrator should guarantee privacy-preservation, for example with the application of federated learning and the transfer of noisy weights. However, it needs to be considered that also obfuscated gradients may become subject to reconstruction attacks and leak information^71,72^.
9. *Resilient to data drift*. XAI approaches need to be evaluated and validated on multicenter datasets to ensure their generalization and robustness against different scanner vendors, imaging protocols, and other potential differences that can cause model drift. In the case of the XAI orchestrator, a model drift can yield an explanation drift that can underscore what the underlying AI systems use as data information to operate. For example, certain XAI saliency maps normalize their internal representation of the data, while others do not. These differences in XAI methods can lead to inconsistencies in XAI results across participating centers where different data acquisition protocols and vendors are used. Here, an interesting area of further research is the development of domain adaptation strategies for XAI technologies.
10. *Up to date*. The pre-trained medical knowledge base should be kept up to date by regular auto-updating.

For the XAI orchestrator to find clinical use, it is critical to develop time-effective and user-friendly Human-Machine Interactions (HMI) systems that are tailored to the specific clinical expert using it^73,74^. In this regard, we believe that the properties of being hierarchical and interactive can be useful in designing and testing HMI systems integrating the proposed XAI orchestrator.

### Functionalities

The XAI orchestrator would offer clinically relevant functionalities that support healthcare workers in their daily tasks.

1. *Information fusion*. The XAI orchestrator could aggregate information faster and more comprehensively than a person could.
2. *Task triage*. In the clinical routine, healthcare workers are often overwhelmed with a large number of tasks, and it is not always straightforward which of them needs to be addressed first. The XAI orchestrator could assist in task tracking and triaging beyond the classical triaging of emergency patients and help healthcare workers in all specialties with time management.
3. *Scenario simulation*. Additionally to summarizing patient data and specialty knowledge, the XAI orchestrator could also aid in extrapolating the effects of additional diagnostic tests or treatments. For example, a diagnostic test might be disadvised if the treatment remains the same independent of the test’s outcome.

### Metrics

Measuring the “goodness” of XAI explanations is an area of active research. Recently, XAI toolkits such as Quantus started to provide evaluation metrics for XAI methods. Quantus structures their evaluation metrics into six groups: faithfulness, robustness, localization, complexity, randomization, and axiomatic metrics^42^. For the XAI orchestrator, we imagine similar metric classes, yet the existing libraries need to be expanded and enriched to be suited for the evaluation of LLMs. Evaluations of LLMs are still scarce, and it has been argued that they measure self-consistency rather than actual faithfulness^75^.

## Possibilities for future implementation of the XAI orchestrator

**Fig. 3:**
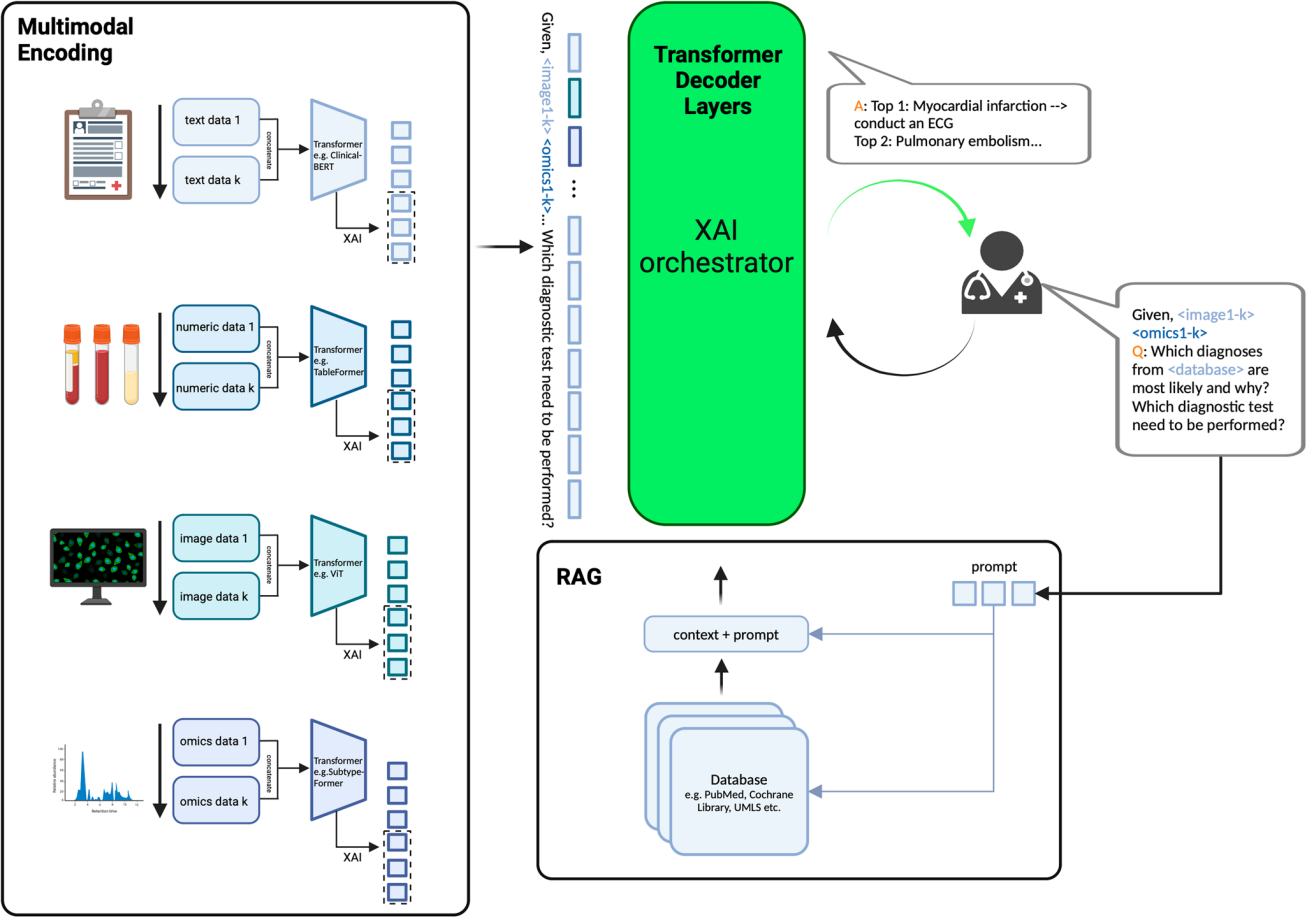
Potential implementation of the XAI orchestrator. Multimodal encodings of patient data, combined with retrieved context information and user prompts feed the decoder, which produces explanations for the user. The user’s question, encoded as a prompt, together with the prior medical knowledge, retrieved e.g. from scientific literature data bases like PubMed are sent to the decoder through retrieval augmented generation (RAG). The central XAI orchestrator decoder is constructed with multiple transformer decoder layers, which generate a textual response to the input question.

Existing transformers can be used to encode the data from different modalities; for example, text data can be processed by Clinical-BERT and images via a vision transformer. The resulting embeddings are concatenated and forwarded jointly to the central XAI orchestrator decoder. The user’s question, encoded as a prompt, together with the prior medical knowledge, retrieved e.g. from scientific literature databases like PubMed, are sent to the decoder through retrieval augmented generation (RAG). The central XAI orchestrator decoder is constructed with multiple transformer decoder layers which generate a textual response to the input question (cf. Fig. 3).

How to answer questions is usually learned from dedicated training data - answers to sample questions that people have phrased specifically for training purposes. This is very time and cost-intensive. As additional training data, verbal interactions like questions and answers that are given by medical professionals during their daily work, for example, during tumor board discussions, could be used. Tumor board session could be recorded and transcribed. These real-world explanations given by medical professionals are likely using highly specific medical vocabulary, as they are intended for colleagues. For a better understanding by the XAI orchestrator, they could be augmented and enriched by another LLM, for example, as in MedKLIP, where a medical knowledge base is queried for entity translation, enabling understanding of unseen entities^76^. Making secondary use of real-world explanations could greatly save time and money and enable training that is closest to the way medical professionals are trained themselves.

## Insights and pathways forward with the XAI orchestrator

XAI methods enjoy rapidly increasing popularity, yet there is still a long way to go to fully transfer the methodological work to clinical implementations. To optimally tailor XAI systems to user needs, clinical domain experts should be involved in the design, development, implementation, and maintenance of (X)AI systems through system development cycles, research partnerships, or advisory roles to facilitate smooth integration into existing workflows, tailoring to the skills and needs of the specific users, and clinical impact^77^. Additionally to medical doctors, this process should involve other clinical professions, like nurses or radiology technicians who may be using the system. Fruitful discussion may be facilitated by clinical experts with solid basic knowledge of the technical aspects of XAI. A need for integration of AI knowledge into core curricula has also been widely expressed among medical students^78^. Next to recommendations from individuals and surveys, which are conducted frequently on various topics in the field of AI^79–83^, a multidisciplinary Delphi study conducted among the targeted user cohort of radiological XAI systems may provide insight into which single solutions most people could agree on. Delphi studies collect expert opinions through questionnaires, just like simple surveys do, but the questionnaires are conducted in multiple rounds, aiming to achieve consensus among the expert group^84^. This is advantageous as the outcome of a Delphi study may provide clearer directions than a simple survey. A recent article describes a Delphi study among experts in the insurance industry to gain insight into their preferences and opinions about XAI^85^. Similar studies concerning radiological XAI applications are currently lacking.

Additionally, educational material adapted to the needs of clinicians is needed. The educational materials on the technical aspect of XAI are often beyond the scope of clinicians’ needs. Materials should focus on the *use*, as opposed to the development, of XAI. Furthermore, it is important to explain to the users what the limitations of a system and its explanations are. For users to trust a system, they need to know over which domain a model is reliable, where it is uncertain, and where it is likely to break down^86^. Changes in explanations need to be observed carefully when the system is confronted with domain changes.

In this review, we aim to bring the attention of the XAI community to the need to develop XAI systems that can handle multimodal and longitudinal data. From analyzing the state of the art on multimodal XAI, we found few studies using XAI methods to produce confirmatory evidence on the good properties of the explained underlying multimodal AI system. Moreover, we observed that these studies remain at a prototype level and encourage the community to further develop and test XAI systems on datasets featuring levels of data quality as found in daily clinical routine. Similarly, various techniques have been proposed to analyze longitudinal data with XAI^57,87–90^, but most have not yet been extensively applied to real-world clinical questions. The critical next step is for these to undergo extensive field testing and external validation. Application to and evaluation on clinical problems should be conducted with the same rigor demonstrated for technical method development. Also, for existing methods, the discussion of what a *good* or reliable explanation constitutes is ongoing (^91^, *inter alia*).

Finally, we propose the “XAI orchestrator” as a virtual assistant to doctors, which is capable of coordinating explanations of specific models and provide a user-centered mechanism to further enquire about AI models operating on multimodal and longitudinal data. With the advent of LLMs and their use in medicine, we believe the development of an LLM-based XAI orchestrator can be a well-timed innovation. However, due to the responsibilities attributed to such a system in coordinating specific (X)AI systems, several challenges still need to be addressed to ensure its reliability, data security, and trustworthiness.

### Reporting summary

Further information on research design is available in the Nature Research Reporting Summary linked to this article.

### Supplementary information

Supplementary material

Reporting Summary

## Data Availability

All studies and other data sources may be accessed through the cited references.

## Code availability

This review article has no associated code base.
