# Supplementary material for: Orchestrating explainable artificial intelligence for multimodal and longitudinal data in medical imaging
Source: NPJ Digit Med. 2024 Jul 22;7:195. doi: 10.1038/s41746-024-01190-w (PMC11263688; doi:10.1038/s41746-024-01190-w)
Supplement: Supplementary file 1 — Supplementary material [file 41746_2024_1190_MOESM1_ESM.docx]

**Supplementary Materials: Clinical Use Case Descriptions**

In clinical practice, the treating clinician receives information from different data modalities at different time points. Some information gives important hints about the correct diagnosis, other information is important in excluding differential diagnoses, and some information is gathered but eventually does not help in finding or excluding the correct diagnosis. The following use case descriptions are entirely fictional and not based on real patients.

**Supplementary Discussion A: Interstitial Pulmonary Fibrosis**

**05.01.2020: Initial Presentation**

*Current complaint (Patient-guided anamnesis):*

Mr. Smith (67y) visits his general practitioner (GP) because of progressive shortness of breath. He recounts that the shortness of breath started six months ago and worsened progressively. He also reports a dry cough and feeling generally fatigued.

*Upon question, the patient reports (Physician-guided anamnesis):*

- Shortness of breath worsens upon exertion.
- The cough is dry, with no hemoptysis.
- No night sweats, weight loss of 2kg in the last six months.
- No chest pains.
- No similar episodes in the past. No history of recurrent atypical pneumonia.
- No travels outside of Europe.
- No one else in the household is sick.
- Exposure to antigens
  - Home: The patient lives in an urban area in an apartment. He did not move to a new home recently. There have not been any water damages within the last years. He does not have a hot tub or sauna. He does not use air conditioning or humidifiers.
  - Workplace: The patient is a retired accountant.
  - Pets: The patient has a dog, which he has been living with since his retirement 5 years ago. No birds.
  - Hobbies: The patient watches sports on TV with his friends and helps his brother in their garden. He has not done any renovations recently and does not play any instruments.
- Signs of connective tissue disease
  - No pruritus or dry skin
  - No ulcerations on fingertips or interphalangeal joints
  - No musculoskeletal pain except for pre-existent lower back pain.
  - No dysphagia, choking, heartburn, or changes of digestion or stool.

*Physical Examination:*

- Vitals: BP 135/86, HR 73, Temp. 36.8°C
- End-expiratory rales over both lungs, Thorax shape is normal.
- Slight bilateral clubbing. Fingers are not swollen.
- No thickening of the skin, no perioral skin tightening.
- No peripheral edema.

*Medical History: The GP consults the patient's file*

- Comorbidities: Atrial fibrillation, hypertension, hip replacement 5y prior, chronic lower back pain
- Medications: Rivaroxaban, Lisinopril, Irfen when needed
- Allergic to avocados and latex.
- Smoked one pack of cigarettes per day from age 20 to 35.

*Laboratory tests are ordered:*

- Hematological tests
- Autoantibodies: anti-nuclear antibody (ANA), anti-centromere antibody (ACA), anti-topoisomerase I antibody (anti-Scl-70), anti-RNA polymerase III antibody, antibodies to Th/To

*Chest XR is acquired in the GP practice:*

- Fine and coarse reticulations, predominantly in the basal lung sections.
- Slightly reduced lung volume.
- No apparent mass, no edema.

**07.01.2020: Laboratory Tests are Back**

*Results from laboratory tests*

- Hematological tests: normal
- Autoantibodies: negative

🡪 The GP sends the patient to the hospital for HRCT and pulmonary function testing.

**17.01.2020: HRCT Findings**

- Honeycombing combined with traction bronchiectasis, reticular opacities, mild ground-glass opacities, light emphysema
- Subpleural and basal predominant
- No pulmonary nodules

🡪 probable UIP pattern

**17.01.2020: Pulmonary Function Testing**

- FVC and TLC reduced
- Preserved expiratory flow rates
- Increased ratio of FEV1/FVC
- Reduced diffusing capacity for carbon monoxide
- Impaired oxygenation

🡪 Consistent with a restrictive defect.

**25.01.2020: Multidisciplinary Discussion**

After the results from the diagnostic tests are in, a multidisciplinary team, including pneumologists and radiologists, meets to discuss. Considering all information jointly, they issue the diagnosis of idiopathic pulmonary fibrosis.

**Summary**

|  | Text-based data | Tabular data | Image data |
| --- | --- | --- | --- |
| 05.01.2020 | - patient history  - information form patient file  - physical examination | - vitals | - CXR |
| 07.01.2020 |  | - laboratory values |  |
| 17.01.2020 |  | - pulmonary function test | - HRCT |
| 25.01.2020 | Multidisciplinary Discussion 🡪 Diagnosis is made. | | |

*Possible model prediction output*

The most likely diagnosis is IFP/UIP. (Combined with a percentage of likelihood).

*Possible Interpretability output*

- For text data: keywords/sentences, for example: “shortness of breath”, “dry cough”, “former smoker”, “exposition to antigens unlikely”, “no signs of connective tissue disease”
- Tabular: Rheumatological serologies negative
- Image: Saliency map highlighting the regions of interest with probable UIP pattern

*
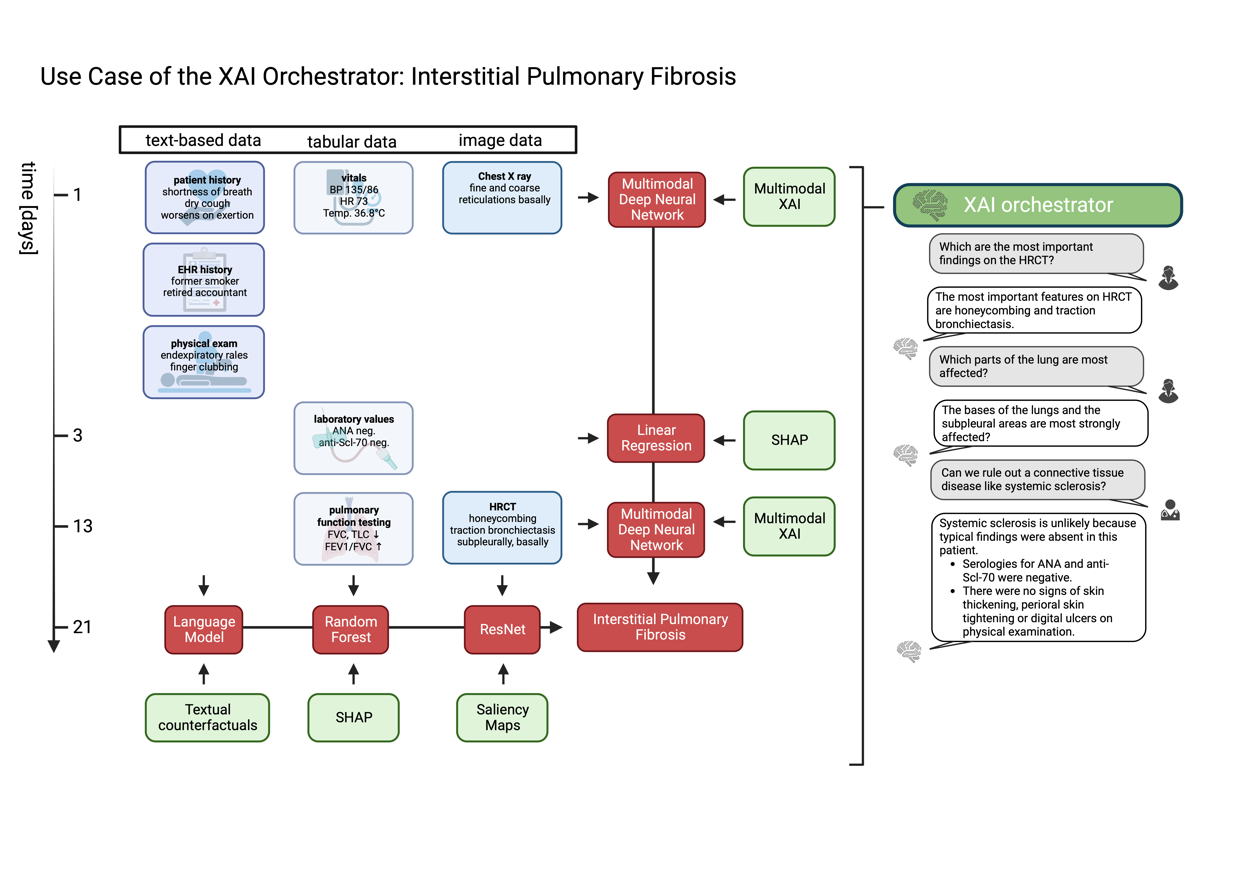
*

*Supplementary Figure 1: The XAI orchestrator scheme applied to the clinical use case of Interstitial Pulmonary Fibrosis. The grid of data points, applied models, and XAI methods follows the general outline in Figure 2 of the main manuscript. Models and XAI methods mentioned are representative examples of suitable popular techniques and can be replaced by other methods depending on the tasks and preferences of the implementing person. In the right part of the figure, a possible interaction between the XAI orchestrator and users, such as radiologists, general practitioners, and pathologists, is proposed. This interaction may include questions about more detailed explanations and counterfactual information. Figure created with BioRender.com.*

**Supplementary Discussion B: Prostate Cancer**

**12.06.2021: Initial Presentation**

Mr. Anderson (58y) has been coming to his GP for yearly check-ups since his 50^th^ birthday. The check-up includes a measurement of prostate-specific antigen (PSA), as Mr. Anderson has a familial disposition for prostate cancer (his father and his brother both had prostate cancer).

Upon question, Mr. Anderson reports lower urinary tract symptoms indicative of prostatic enlargement: Over the last years, he noticed an increase in urinary frequency during the day and reports nocturia 2x/night, with low International Prostatism Symptom Score (IPSS) of 5. He also reports slowing of the urinary stream and urinary hesitancy.

Mr. Anderson has no co-morbidities and does not take any regular medication.

Throughout the last years, PSA was measured to be within the upper range of normal, with a slight increase over the years. (Range 2.7 – 3.5ng/ml) This year, PSA is 4.9ng/ml.

The exceeding of 4ng/ml, as well as the increase of >0.75ng/ml within one year, warrant further investigation. Mr. Anderson is asked to come to the GP’s office again two weeks later to repeat the measurement to confirm the PSA value.

**26.06.2021: Repeat PSA Measurement**

PSA is measured to be 5.0ng/ml.

The digital rectal examination is inconclusive.

The GP refers Mr. Anderson to a urologist.

**01.07.2021: First Urologist Consultation with Transrectal Ultrasound**

The urologist performs a transrectal ultrasound, which shows an enlarged prostate (60ml) with hyperplastic nodules as well as a hypoechoic lesion.

He refers Mr. Anderson to the hospital for an MRI.

**08.07.2021: MRI**

An MRI is acquired showing:

- Total prostate volume enlarged, 63ml.
- Lesion of 1.2cm in the transition zone, T2 and ADC hypointense, hyperintense on high b-value DWI in the transition zone. No extracapsular extension and no infiltration of the seminal vesicle.
- Enlarged central gland with heterogeneous signal.

🡪 Lesion with PI-RADS 4 and prostate hyperplasia

**15.07.2021: Biopsy**

A systematic biopsy is performed and the tissue samples are sent to pathology.

- small, cuboidal cells
- Variable gland architecture, stromal invasion, and individual glands can still be identified.
- prominent nucleoli

🡪 prostatic adenocarcinoma of the acinar type, Gleason score 3+4, no extracapsular extension, no seminal vesical invasion, no vascular or perineural invasion

**20.07.2021: Discussion of Results and Treatment Planning**

The urologist explains to Mr. Anderson that he has a localized stage of prostate of intermediate risk. Treatment options are explained and after discussions about the advantages and disadvantages of treatment options, Mr. Anderson expresses his preference to undergo surgery. Considering Mr. Andersons cancer characteristics, his prior lower urinary tract symptoms, his treatment preference, as well as the fact that Mr. Anderson had no prior abdominal surgeries, the patient and urologist agree to surgical therapy.

**Summary**

|  | Text-based data | Tabular data | Image data |
| --- | --- | --- | --- |
| 12.06.2021 | - lower urinary tract symptoms: increased frequency, nocturia, hesitancy  - family history of prostate cancer  - information from the patient file: no prior abdominal surgeries | PSA |  |
| 26.06.2021 | physical examination | PSA |  |
| 01.07.2021 |  |  | TRUS |
| 08.07.2021 |  |  | MRI |
| 15.07.2021 |  |  | WSI |
| 20.07.2021 | Urologist makes a diagnosis. | | |

*Possible Model Prediction output*

The most likely diagnosis is prostatic adenocarcinoma, Gleason 3+4.

*Possible Interpretability output*

Text data: keywords/sentences, for example:

- family history of prostate carcinoma
- lower urinary tract symptoms

Tabular: PSA 5.0ng/ml

Image: TRUS: saliency map, highlighting the lesion

MRI: saliency map, highlighting the lesion

Pathology: saliency map highlighting the irregular glands

*
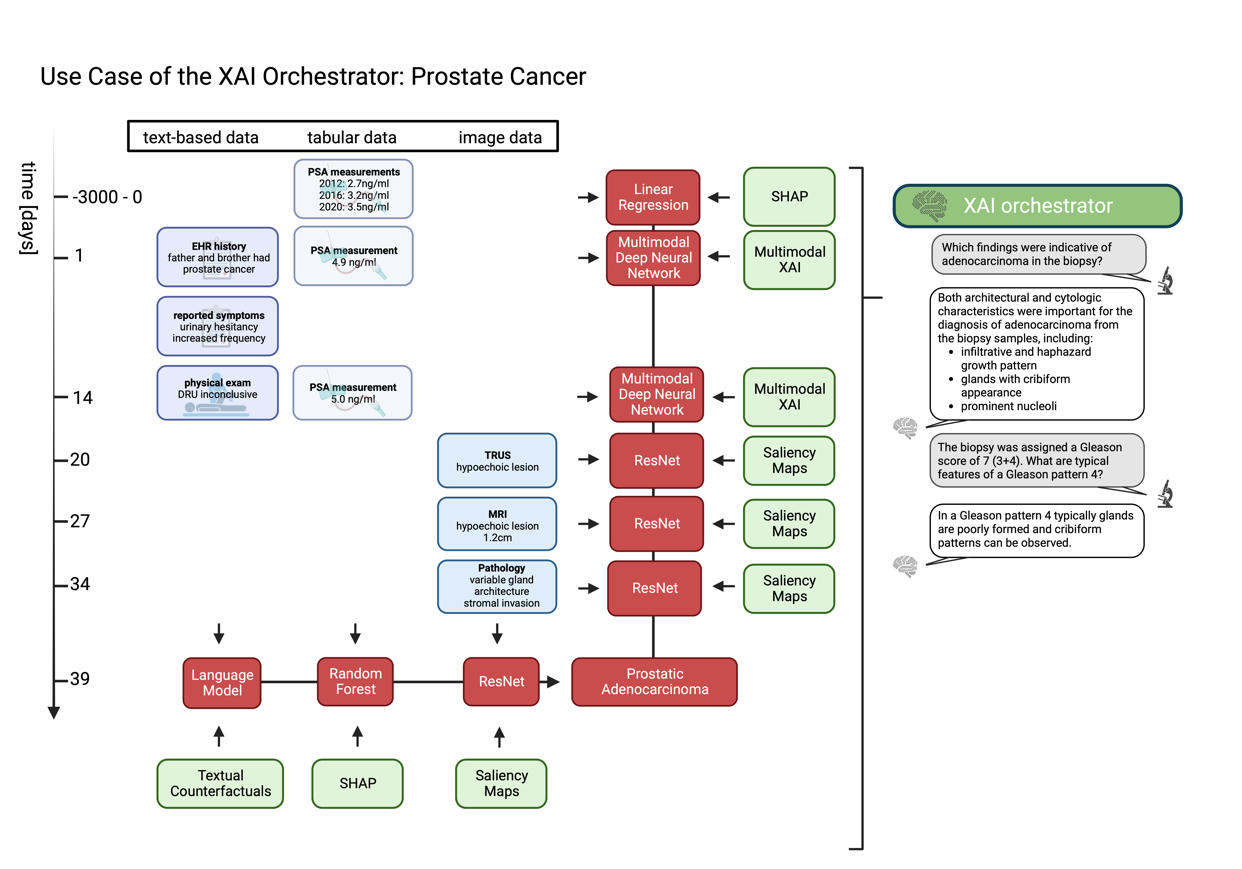
*

*Supplementary Figure 2: The XAI orchestrator scheme applied to the clinical use case of Prostate Cancer. Corresponding to Supplementary Figure 1, the grid of data points, applied models, and XAI methods follows the general outline in Figure 2 of the main manuscript, and the models and XAI methods mentioned are representative examples of suitable popular techniques. Figure created with BioRender.com.*
